# Chemical Stability and Leaching Behavior of ECO EPDM in Acidic Fuel Cell-like Conditions

**DOI:** 10.3390/ma18143260

**Published:** 2025-07-10

**Authors:** Daniel Foltuț, Georgiana-Iulia Șoșoi, Viorel-Aurel Șerban

**Affiliations:** 1 Department of Materials and Manufacturing Engineering, Politehnica University of Timișoara, Mihai Viteazul Blv., 300222 Timisoara, Romania; viorel.serban@upt.ro; 2 Department of Applied Chemistry, Organic and Natural Compounds Engineering, Politehnica University of Timișoara, Carol Telbisz 6, 300001 Timisoara, Romania; georgiana.sosoi@student.upt.ro; 3 Technical Sciences Academy of Romania, B-dul Dacia 26, 030167 Bucharest, Romania

**Keywords:** EPDM elastomer, circular carbon black, recycled carbon black, chemical aging, ionic leaching, PEM fuel cell, hot water extraction, HPLC-DAD analysis, surface degradation, sustainable materials

## Abstract

This study investigates the chemical stability and leaching behavior of two environmentally sustainable EPDM elastomers filled with circular carbon black (CCB) and recycled carbon black (RCB) when exposed to acidic, fuel cell-like environments. Accelerated aging tests were conducted in sulfuric acid solutions of varying concentrations (1 M, 0.1 M, and 0.001 M) at 90 °C for 1000 h to simulate long-term degradation in proton exchange membrane fuel cell (PEMFC) sealing applications. Complementary hot water extraction tests (HWET) were performed at 80 °C for up to 168 h to evaluate ionic leaching via conductivity measurements. HPLC-DAD analysis was used to assess organic leachates, while surface changes were examined by SEM and thermal transitions by DSC. Results revealed lower leaching and improved surface preservation in the CCB-filled EPDM, which remained below the critical 5 µS/cm ionic conductivity threshold for longer durations than its RCB counterpart. HPLC results showed filler-dependent trends in organic compound release, with CCB EPDM exhibiting higher leaching only under strong acid exposure. SEM confirmed greater surface damage and porosity in RCB EPDM. Overall, both materials demonstrated adequate chemical resistance, but the CCB formulation exhibited superior long-term stability, supporting its use in sustainable PEMFC sealing applications.

## 1. Introduction

Proton exchange membrane fuel cells (PEMFCs) have emerged as promising power sources in transportation and stationary applications due to their high efficiency, low emissions, and rapid startup characteristics [1,2,3]. In these systems, sealing materials play a critical role in ensuring gas isolation, maintaining mechanical compression, and preventing leaks of hydrogen and oxygen [4,5,6,7]. The performance and longevity of PEMFCs are directly influenced by the durability of these seals, which must endure high temperatures (60–90 °C), humidity, and chemical exposure, particularly to acidic byproducts such as sulfuric acid and fluoride species formed during operation [8].

Ethylene propylene diene monomer (EPDM) rubber is widely used in PEMFC sealing applications due to its resistance to polar media, thermal stability, and low compression set [9,10]. To reduce the environmental impact of conventional rubber production, sustainable variants of EPDM have been developed, incorporating circular carbon black (CCB) and recycled carbon black (RCB) as fillers. CCB is sourced from closed-loop carbon black processes, while RCB is derived from waste tire pyrolysis [11,12,13]. These approaches aim to reduce fossil-derived carbon input while promoting material circularity and waste reduction. The behavior of recycled carbon black (RCB) in elastomeric systems has been the subject of recent studies exploring its performance and limitations. Rikmann et al. [11] demonstrated that low-quality RCB derived from tire pyrolysis often contains residual organics and polyaromatic hydrocarbons (PAHs), which reduce the surface area and promote particle agglomeration. However, post-processing via cavitational vortex milling was shown to improve the purity and dispersibility of RCB, highlighting the potential to tailor surface characteristics through additional treatment. Billotte et al. [12] examined HDPE composites containing 6 wt.% RCB and found that RCB significantly enhanced UV protection, reducing photon penetration by up to 54% compared to virgin HDPE, while also contributing to oxidative stability under aging. Ghosh et al. [13] further showed that incorporating reinforcing-grade carbon black into devulcanized rubber composites could improve tensile strength by up to 15%, though high filler loading adversely affected elongation and abrasion resistance. These findings collectively emphasize the complex balance between the structural advantages and chemical reactivity of recycled fillers—insights that are directly relevant to the performance disparity observed between CCB and RCB EPDM in the present work.

Despite its chemical resilience, EPDM is not immune to degradation in fuel cell environments. Prolonged exposure to heat and acid can induce oxidative chain scission, filler–matrix separation, and leaching of ions or additives. A degradation rate-based model developed by Zaghdoudi et al. [14] demonstrated that stress relaxation in EPDM during thermal aging involves two distinct chemical processes, each governed by different activation energies, with lower-energy processes dominating early-stage degradation. This modeling approach, validated using Arrhenius and time–temperature superposition techniques, emphasized the importance of distinguishing between physical and chemical relaxation contributions for accurate lifetime prediction of EPDM seals. Complementarily, Zhao et al. [15] highlighted that EPDM remains a widely used material for PEMFC sealing applications due to its flexibility and resistance to heat and oxidation; however, they also noted that harsh fuel cell environments—characterized by high temperature, humidity, and acidic condensates—necessitate precise material selection and continuous performance evaluation to ensure sealing integrity. Thermal and acidic aging may affect not only bulk mechanical properties but also surface integrity, leading to increased permeability and reduced sealing performance [8,16]. The introduction of recycled fillers like RCB may exacerbate these effects due to the presence of residual organics and altered filler morphology, potentially compromising long-term stability [11,13,17].

Thermoset EPDM rubber cannot be remelted due to its irreversible crosslinked network structure. Recent developments in devulcanization techniques, such as continuous thermomechanical processing using twin-screw extrusion, have achieved up to 93.9% devulcanization efficiency, particularly under optimized thermal and mechanical conditions. However, high devulcanization rates can be offset by increased random scission, which reduces the mechanical integrity of the recycled material. Careful control of process parameters is therefore required to preserve material quality suitable for PEMFC gaskets [17]. Simultaneously, bio-based EPDM formulations have emerged through the use of renewable monomers such as bio-derived ethylene and propylene, sourced from biomass fermentation and dehydration of ethanol. Although major tire manufacturers have begun incorporating bio-isoprene in their rubber compounds, the overall market share of biomass-derived chemicals in rubber production was still only 2.6% in 2020, underlining the early stage of this technology’s industrial adoption. Challenges such as production cost, process optimization, and mechanical property equivalence limit their current application in PEMFC seals [16].

Previous investigations by the authors [18,19] established the mechanical and physicochemical degradation behavior of conventional EPDM, TPV, and ECO TPV under thermal and acidic aging conditions relevant to PEMFC environments. One study [19] focused on evaluating tensile properties and compression set behavior after exposing the materials to sulfuric acid solutions (1 M, 0.1 M, and 0.001 M) and thermal aging at 90 °C for 1000 h. Among the tested materials, EPDM exhibited the highest tensile strength retention and the lowest compression set values, indicating strong elasticity and resistance to long-term deformation. TPV and ECO TPV showed greater changes in elongation and stress responses, particularly under chemical exposure. Notably, the pH of immersion solutions increased after aging, most significantly for EPDM, suggesting leaching or buffering activity at the rubber–solution interface. In a complementary study [18], the same materials were analyzed using more advanced characterization techniques, including volume and weight change analysis, DSC, Shore A and micro-hardness testing, SEM, and hot water ion extraction tests. The results confirmed that EPDM had the lowest mass and volume changes, minimal DSC shift, and moderate ion leaching, whereas ECO TPV showed substantial deterioration. The conductivity of immersion water in the hot water extraction test exceeded 8.0 µS/cm for ECO TPV, indicating significant ionic mobility or release, while EPDM reached 3.9 µS/cm after 100 h. SEM micrographs revealed surface cracking and erosion in ECO TPV and TPV after acid and thermal aging, while EPDM retained a smoother morphology. These studies provided a robust baseline demonstrating EPDM’s superior chemical and thermal stability and helped identify ECO TPV as particularly vulnerable. However, neither study included detailed organic leachate analysis or examined differences between CCB and RCB filler systems—gaps the present work addresses through HPLC-DAD, pH tracking, and DSC comparison of acid- and heat-aged materials.

Building upon these foundational results, the authors recently published a detailed analysis focused on CCB- and RCB-filled EPDM compounds [20]. The study evaluated mechanical performance degradation after thermal and acidic aging in 1 M, 0.1 M, and 0.001 M H_2_SO_4_ solutions at 90 °C for 1000 h. CCB EPDM demonstrated superior dimensional stability, retaining over 90% of its tensile strength and exhibiting minimal swelling (e.g., 0.97% volume increase at 1 M). In contrast, RCB EPDM showed more extensive degradation, with swelling exceeding 4.5% and tensile strength retention as low as 68% in some conditions. SEM-EDS analysis revealed that RCB EPDM surfaces developed more pronounced porosity, oxidation, and sulfur uptake, while CCB EPDM retained a smoother morphology and lower elemental migration. These findings supported the enhanced environmental resilience of CCB-filled EPDM and identified RCB filler morphology and chemical activity as key contributors to degradation.

To date, no study has systematically examined how these eco-sustainable EPDM formulations respond to long-term acidic immersion from a chemical leaching perspective. The present study builds directly on this previous work by shifting the focus from mechanical degradation to chemical stability and leaching behavior. In addition to confirming dimensional and surface stability, it aims to address unresolved questions regarding acid–polymer interaction mechanisms, pH changes, ion migration, and organic leachate formation. To this end, both CCB and RCB EPDM samples were subjected to immersion in 1 M, 0.1 M, and 0.001 M H_2_SO_4_ for 1000 h at 90 °C. New characterization methods include the following:

- Tracking of initial and final pH values to assess acid consumption or neutralization;
- Conductivity measurements of hot water extracts to evaluate ionic leaching;
- Differential scanning calorimetry (DSC) to compare thermal transitions of fresh, heat-aged, and acid-aged materials;
- High-performance liquid chromatography with diode array detection (HPLC-DAD) on dried aging solutions to examine the presence of UV-active leachates.

In addition, new high-magnification SEM images are evaluated to assess changes in surface microstructure related to acid exposure. This comprehensive approach offers new insights into the filler-dependent chemical durability of sustainable EPDMs in environments simulating long-term PEMFC operation.

## 2. Materials and Methods

### 2.1. Materials

Two peroxide-cured EPDM elastomer compounds were investigated in this study: one formulated with circular carbon black (CCB EPDM) and the other with recycled carbon black (RCB EPDM). Both materials were manufactured by Arlanxeo (Geleen, The Netherlands) and supplied as vulcanized sheets. The matrix polymer and curing system were held constant to isolate the effect of filler type on degradation and leaching behavior. The CCB filler was derived via advanced pyrolysis and refinement of end-of-life tires, while the RCB filler was obtained through conventional pyrolytic recycling, with less controlled particle morphology and potential surface residue variability. The mechanical properties of the fresh materials are listed in Table 1.

### 2.2. Aging Protocols

Aging procedures were designed to simulate harsh conditions typical of proton exchange membrane fuel cell (PEMFC) environments. Each EPDM type was subjected to the following:

- Chemical aging: Immersion in DI water and H_2_SO_4_ solutions at concentrations of 1 M, 0.1 M, and 0.001 M.
- Thermal aging: Exposure to dry heat at 90 °C for 1000 h.

For chemical aging, EPDM specimens were placed in sealed borosilicate glass containers filled with 50 mL of acid solution and stored in a temperature-controlled oven at 90 °C for 1000 h. After aging, samples were rinsed with deionized water, air-dried, and stored in a desiccator. Thermal aging followed the same temperature and duration but without immersion.

### 2.3. pH Tracking and Hot Water Extraction Test

The pH of the acid solutions was measured before and after aging using a calibrated Mettler Toledo FiveEasy™ Plus pH meter (Mettler Toledo, Greifensee, Switzerland). Each reading was performed at room temperature (23 ± 1 °C), and solutions were analyzed immediately after sample removal to minimize environmental exposure.

The samples were taken out from a 2 mm sheet, resulting in a total surface area of 23.2 cm^2^.

To evaluate ion leaching under neutral conditions, a hot water extraction test was conducted. CCB and RCB EPDM samples were immersed in 100 mL of deionized water at 80 °C for 168 h. The water conductivity before and after immersion was measured using an HI98308 PWT tester (Hanna Instruments, Woonsocket, RI, USA), with a working range of 0.0 to 99.9 µS/cm and a resolution of 0.1 µS/cm. The goal was to evaluate the extent of ionic species leaching from the EPDM samples into the solution, complementing SEM-EDS by detecting soluble, non-surface-bound ions that may not be visible through surface analysis.

### 2.4. High-Performance Liquid Chromatography (HPLC) Analysis

Chromatographic analysis of organic compounds leached from the EPDM samples after immersion in 1 M, 0.1 M, and 0.001 M H_2_SO_4_ for 1000 h at 90 °C was carried out using high-performance liquid chromatography (HPLC). The system used was an Agilent 1260 Infinity II, equipped with a UV–Vis diode array detector (DAD). The instrument was manufactured by Agilent Technologies (Santa Clara, CA, USA), with the relevant production facility for HPLC equipment located in Waldbronn, Baden-Württemberg, Germany. Chromatographic separation was performed on a Teknokroma C18 reversed-phase column (Teknokroma Analítica S.A., Barcelona, Spain).

Prior to injection, each solution was filtered using a 0.45 μm syringe filter to remove solid particles. A dilution step was performed by mixing 100 μL of the original sample with 900 μL of acetonitrile, resulting in a final volume of 1 mL per sample.

The mobile phase consisted of acetonitrile and water, applied in a gradient elution regime followed by an isocratic phase. The flow rate was maintained at 1.0 mL/min, and the injection volume was set to 20 μL for all measurements.

Detection was carried out at multiple wavelengths (220, 230, 240, 250, and 270 nm) to optimize the identification of UV-active leachates. All chromatographic runs were conducted at a controlled temperature of 25 °C to ensure reproducibility and consistency across measurements.

### 2.5. Differential Scanning Calorimetry (DSC)

Thermal transitions of the fresh and aged EPDM materials were evaluated by differential scanning calorimetry (DSC). The measurements were conducted using a Discovery DSC 25P (TA Instruments, New Castle, DE, USA), equipped for high-pressure analysis and nitrogen purging.

Each analysis was performed under a nitrogen atmosphere to prevent oxidative degradation during heating. Nitrogen gas was supplied at a constant purge flow rate of 40 mL/min to ensure an inert environment within the sample chamber.

The temperature program was initiated at −60 °C and ramped up to 200 °C at a constant heating rate of 10 K/min. This range was selected to encompass potential glass transition phenomena and other thermal events relevant to the elastomeric matrix and its fillers. The tests were conducted on samples in fresh, thermally aged, and acid-aged conditions to assess shifts in thermal behavior that could indicate material degradation or structural modification.

### 2.6. Scanning Electron Microscopy and Energy-Dispersive X-Ray Spectroscopy (SEM-EDS)

The surface morphology and microstructural degradation of the EPDM samples were investigated using scanning electron microscopy (SEM) coupled with energy-dispersive X-ray spectroscopy (EDS). Fresh, thermally aged, and acid-aged (1 M H_2_SO_4_, 1000 h) specimens of both CCB and RCB EPDM were analyzed to assess changes in surface integrity, filler distribution, and elemental migration.

SEM imaging was performed using a Thermo Fisher Axia ChemiSEM system (Thermo Fisher Scientific, Waltham, MA, USA). Samples were mounted on aluminum stubs using conductive carbon adhesive and sputter-coated with gold to ensure surface conductivity. Images were acquired using a CBS detector at an accelerating voltage of 10.00 kV and a working distance of approximately 10.2 mm. Micrographs were captured at 500× and 1000× magnification, with the horizontal field width (HFW) for 1000× set to ~414 µm, enabling detailed assessment of surface topography, including porosity, filler–matrix interaction, and degradation features.

Elemental mapping and point analysis were conducted using the system’s integrated EDS detector. Spectra were acquired at 10.00 kV for a total acquisition time of 379 s, with an average count rate of ~1140 cps and total counts exceeding 430,000. EDS maps were recorded at a resolution of 768 × 512 pixels. Quantitative results were reported in both atomic and weight percent, with a focus on key elements such as carbon (C), oxygen (O), and sulfur (S) and trace components including magnesium (Mg), silicon (Si), and zinc (Zn). Changes in sulfur and oxygen content after acid exposure were used to infer the extent of oxidative- and acid-induced degradation, while Mg and Zn were tracked as potential indicators of filler leaching or residue from recycled content.

The SEM-EDS results were used to correlate observed surface morphology with aging mechanisms and filler-dependent stability trends.

## 3. Results

### 3.1. pH Tracking and Hot Water Extraction Test Results

To evaluate the chemical interaction between EPDM materials and acidic environments, the pH values of sulfuric acid solutions were recorded before and after 1000 h of immersion at 90 °C. Three concentrations were tested: 1 M, 0.1 M, and 0.001 M H_2_SO_4_. The initial pH values of the immersion solutions were measured as 0.10, 0.84, and 2.24, respectively, in agreement with previously reported data [19].

After exposure, notable changes in pH were observed depending on the material type and acid concentration, as seen in Table 2. For the EPDM with circular carbon black (CCB), a decrease in pH was observed across all conditions, indicating possible retention or release of acidic species. In contrast, the EPDM containing recycled carbon black (RCB) exhibited pH increases in all tested solutions, suggesting leaching of basic or buffering components that neutralized some of the acidity.

To assess the leaching of ionic species from the elastomer matrix under neutral conditions, a hot water extraction test (HWET) was performed following the protocol described in Section 2.3. The conductivity of the extraction solution was measured at regular intervals (0 h, 24 h, 48 h, 96 h, and 168 h) to monitor the release of water-soluble compounds from the EPDM samples.

**Figure 1:**
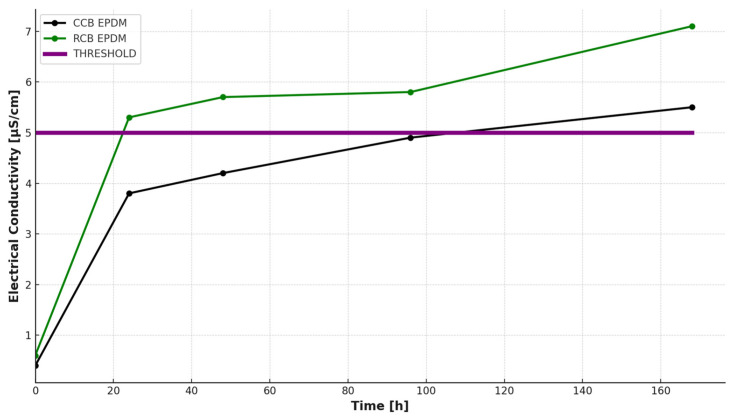
Electrical conductivity of hot water extracts from CCB and RCB EPDM samples measured over a 168 h period at 80 °C.

The initial conductivity of the deionized water was low for both materials (0.4 μS/cm for CCB and 0.6 μS/cm for RCB). Upon immersion at 80 °C, the conductivity values increased steadily over time, as shown in Figure 1. After 168 h, the conductivity reached 5.5 μS/cm for the CCB sample and 7.1 μS/cm for the RCB sample, indicating higher ionic release from the RCB formulation.

The RCB EPDM showed a sharper increase in conductivity within the first 48 h, crossing the 5 μS/cm threshold [21] by the 48 h mark. In contrast, CCB EPDM reached this threshold only after 96 h. This behavior suggests a higher level of extractable ionic or polar species in the RCB formulation, likely due to residual contaminants or processing by-products from the recycled carbon black.

Such differences in ionic leaching have important implications for fuel cell applications, where the conductivity of adjacent materials and exposure to moisture can influence long-term performance and degradation. The faster and higher release of ions from RCB EPDM may impact membrane contamination and local acid–base interactions or even promote corrosion of adjacent components.

Overall, the HWET results complement the pH tracking analysis by providing a time-resolved profile of ionic mobility under water exposure, reinforcing the observation that RCB-based formulations are chemically less stable or more reactive in aqueous environments compared to their CCB counterparts.

### 3.2. Differential Scanning Calorimetry Results

Differential scanning calorimetry (DSC) was employed to assess potential changes in the thermal behavior of the ECO EPDM samples after chemical aging in 1 M H_2_SO_4_ at 90 °C for 1000 h.

**Figure 2:**
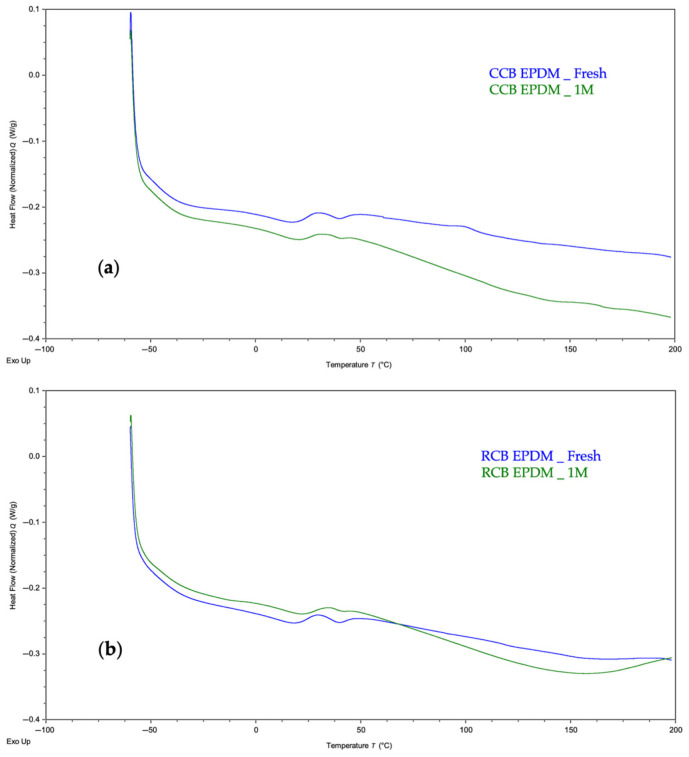
DSC thermograms of CCB EPDM (**a**) and RCB EPDM (**b**) before and after chemical aging in 1 M H_2_SO_4_ at 90 °C for 1000 h.

For both materials, the primary thermal transition observed was the glass transition temperature (Tg), which is a critical indicator of segmental mobility in the elastomer matrix. Representative thermograms comparing the fresh and acid-aged samples are shown in Figure 2a (CCB EPDM) and Figure 2b (RCB EPDM).

The fresh CCB EPDM exhibited a Tg of approximately –49.1 °C, which remained nearly unchanged (−48.9 °C) after acid exposure, suggesting that the polymer network and filler interaction remained stable during aging. The shape and baseline shift of the curve were also similar before and after exposure, indicating minimal plasticization or degradation.

In contrast, RCB EPDM showed a slight downward shift in Tg from −47.3 °C (fresh) to −48.5 °C (aged), as depicted in Figure 2b. This change, although subtle, may indicate minor polymer chain scission or migration of low-molecular-weight components from the matrix. The slight reduction in thermal stability for RCB EPDM aligns with the higher ionic leaching observed in the HWET and pH tracking tests, potentially reflecting greater degradation at the molecular level.

In addition to the main glass transition (Tg) observed between −45 °C and −50 °C, both EPDM formulations exhibited minor endothermic features between 20 and 50 °C. These are likely related to the melting of low-molecular-weight waxes or processing aids used in compounding. Such transitions are common in filled elastomers and do not represent major phase changes, but they may indicate partial migration or redistribution of surface-active additives during aging. [22]

Overall, the DSC data confirm that both materials retain their basic thermomechanical stability upon exposure to acidic conditions. However, the observed trends suggest that the circular carbon black (CCB)-based formulation exhibits slightly higher thermal stability under aggressive chemical aging than the recycled carbon black (RCB) formulation.

### 3.3. HPLC-DAD Analysis

To evaluate the migration of organic compounds from the EPDM samples into acidic aqueous media, high-performance liquid chromatography with diode array detection (HPLC-DAD) was performed at 230 nm. The analysis focused on the comparison between CCB- and RCB-filled EPDM after aging in sulfuric acid solutions of varying concentrations (0.001 M, 0.1 M, and 1 M) for 1000 h at 90 °C. The chromatograms and corresponding peak areas provide insight into the extent and profile of leachable substances as a function of material formulation and chemical exposure.

**Figure 3:**
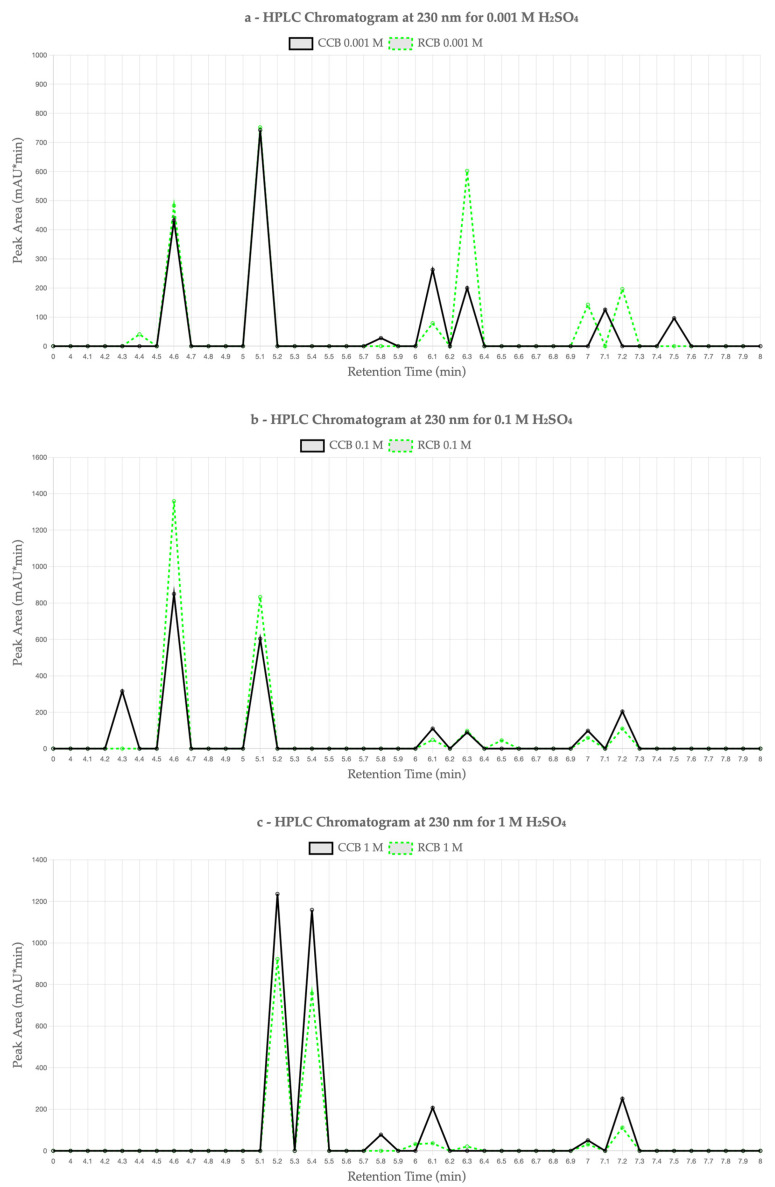
HPLC chromatograms at 230 nm for CCB and RCB EPDM samples extracted in (**a**) 0.001 M H_2_SO_4_, (**b**) 0.1 M H_2_SO_4_, and (**c**) 1 M H_2_SO_4_ for 1000 h at 90 °C. Solid black lines represent CCB and dashed green lines represent RCB.

Figure 3 presents chromatograms of the leachates at 230 nm for each acid concentration: (a) 0.001 M, (b) 0.1 M, and (c) 1 M H_2_SO_4_. In each subplot, solid lines represent the CCB-based EPDM samples, while dashed lines correspond to the RCB formulations. Across all concentrations, distinct differences are observed in both the number and intensity of peaks, indicating material-dependent leaching behavior.

At 0.001 M H_2_SO_4_ (Figure 3a), RCB EPDM exhibited a higher number of peaks and greater total area than CCB, with particularly prominent peaks at retention times (RTs) of 4.651 min (482.73 mAU·min), 5.131 min (751.70 mAU·min), and 6.357 min (602.13 mAU·min). The CCB sample showed notable peaks at 4.661 min (431.85 mAU·min), 5.135 min (742.46 mAU·min), and 6.137 min (262.46 mAU·min), but with lower cumulative intensity.

At 0.1 M H_2_SO_4_ (Figure 3b), both samples showed increased total leaching compared to the 0.001 M case. The RCB sample again showed the most intense peaks—particularly at 4.658 min (1358.91 mAU·min) and 5.138 min (833.13 mAU·min). CCB also displayed elevated peak areas, notably at 4.652 min (850.26 mAU·min) and 5.134 min (603.79 mAU·min), though overall peak intensities remained lower than those of RCB.

In contrast, at 1 M H_2_SO_4_ (Figure 3c), CCB exhibited stronger leaching than RCB, with dominant peaks at 4.685 min (1235.30 mAU·min) and 5.262 min (1158.85 mAU·min), yielding a higher total area (2980.14 mAU·min) than the RCB sample (1910.97 mAU·min), whose main peaks occurred at 4.683 min (922.12 mAU·min) and 5.250 min (757.07 mAU·min). This inversion in leaching intensity suggests that at high acidity, the CCB matrix may become more susceptible to additive or filler migration.

Interestingly, the total peak area for RCB EPDM decreased at the highest acid concentration (1 M) compared to the lower concentrations of 0.1 M and 0.001 M. This counterintuitive result may be attributed to the breakdown of extractable organic compounds into lower-molecular-weight fragments that are either non-UV-active or fall below the detection threshold of the DAD at 230 nm. Additionally, prolonged exposure to strong acid could lead to surface passivation or structural densification of the elastomer matrix, which may restrict further migration of leachable species. Similar effects have been observed in polymer degradation studies, where increased chemical aggressiveness does not necessarily correlate with higher extractable content due to matrix collapse or secondary degradation phenomena [23,24]. These findings underscore the complexity of leaching mechanisms in filled elastomers and highlight the need for more advanced analytical techniques, such as mass spectrometry, to more precisely identify the degradation products [18,19].

Table 3 summarizes the HPLC-DAD results, detailing the total peak area and the number of peaks exceeding 100 mAU·min for each material and acid concentration. The data reveals that, while the RCB EPDM tends to release more organic compounds at lower acid concentrations, the CCB EPDM shows a higher degree of leaching at high acid concentrations, pointing to potential filler and additive migration mechanisms in both systems.

**Figure 4:**
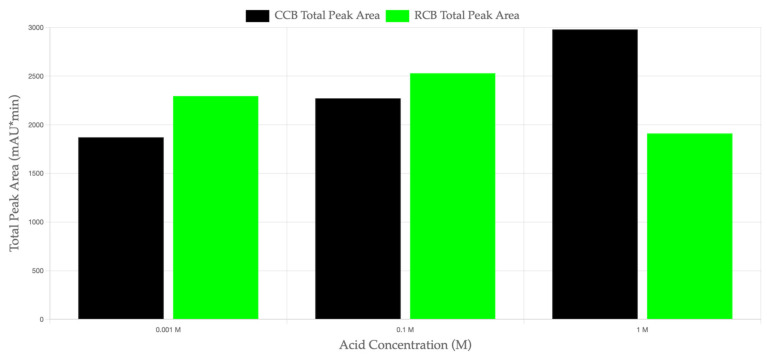
Total peak area (mAU·min) of leachates from CCB and RCB EPDM samples in sulfuric acid solutions of varying concentrations (0.001 M, 0.1 M, and 1 M for 1000 h at 90 °C). The bars represent the total peak areas recorded by HPLC-DAD at 230 nm, with the black bars corresponding to CCB EPDM and the green bars representing RCB EPDM.

The HPLC-DAD analysis revealed distinct differences in the total peak area between CCB and RCB EPDM materials exposed to sulfuric acid solutions of varying concentrations (0.001 M, 0.1 M, and 1 M). Figure 4 illustrates the total peak area (mAU·min) for both materials across the different acid concentrations. At lower acid concentrations (0.001 M and 0.1 M), RCB EPDM exhibited a higher total peak area, indicating a greater leaching of organic compounds from the recycled carbon black formulation. This suggests that RCB EPDM is more prone to leaching under mild acidic conditions, likely due to the residual contaminants and unreacted components in the recycled filler.

In contrast, at 1 M H_2_SO_4_, CCB EPDM demonstrated a higher total peak area, indicating a more significant migration of organic compounds in the highly acidic environment. This inversion in leaching intensity at higher acid concentrations suggests that the CCB filler may become more reactive in strongly acidic conditions, possibly due to the increased solubility of the additives or fillers under these conditions. The data in Figure 4 supports the hypothesis that CCB and RCB fillers exhibit different leaching behaviors depending on the acid concentration, highlighting the influence of filler type on the chemical stability of EPDM in acidic environments.

### 3.4. SEM Analisys

To further assess the impact of acidic aging on the surface morphology of CCB and RCB EPDM, scanning electron microscopy (SEM) was employed to observe the microstructure of the samples before and after exposure to sulfuric acid solutions. The images provide crucial insight into filler distribution, surface integrity, and potential degradation features at a magnification of 5000×.

**Figure 5:**
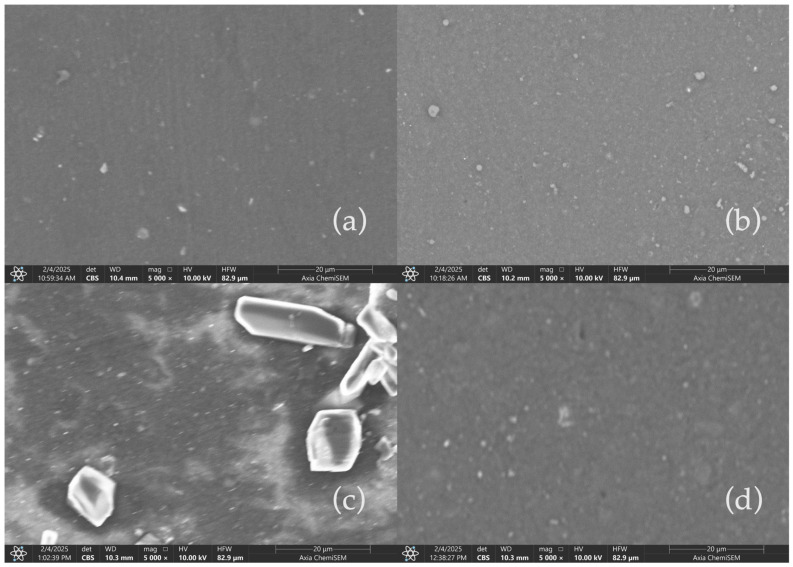
SEM micrographs of CCB and RCB EPDM samples at 5000× magnification. (**a**) Fresh CCB EPDM, showing a smooth surface with minimal irregularities. (**b**) Fresh RCB EPDM, with more visible filler particles and irregular distribution. (**c**) RCB EPDM after exposure to 1 M H_2_SO_4_ for 1000 h, showing significant surface degradation with cracks and porous regions. (**d**) CCB EPDM after 1000 h in 1 M H_2_SO_4_.

Figure 5 presents SEM micrographs of the EPDM materials under fresh (a,c) and acid-aged (b,d) conditions. In the fresh CCB EPDM (a), the surface appears smooth with minimal irregularities, suggesting a well-integrated filler–matrix interaction. Upon exposure to 1 M H_2_SO_4_ for 1000 h (b), the CCB EPDM surface suffers a slight increase in surface roughness.

In contrast, fresh RCB EPDM (c) reveals more visible filler particles, which are more irregularly distributed. After 1000 h of immersion in 1 M H_2_SO_4_ (d), the RCB EPDM shows more significant surface degradation, including the development of noticeable surface cracks and the formation of porous regions. These changes are indicative of the higher chemical reactivity and susceptibility of RCB EPDM to acid-induced degradation, likely due to the residual contaminants and the nature of the recycled carbon black filler [20].

These SEM observations underscore the differences in chemical stability between the CCB and RCB EPDM formulations, aligning with the leaching and pH tracking results. The increased surface damage observed in RCB EPDM after acid aging highlights the challenges associated with using recycled fillers in harsh environments such as those found in fuel cell applications.

## 4. Discussion

The present study provides an integrated analysis of the chemical stability and leaching behavior of sustainable EPDM materials filled with either circular carbon black (CCB) or recycled carbon black (RCB) under simulated fuel cell-like acidic environments. By extending earlier mechanical aging evaluations [20], this work shifts the focus toward chemical degradation pathways, leachate formation, and surface morphological evolution, offering a broader understanding of material suitability for PEMFC sealing applications.

### 4.1. Comparison with Conventional EPDM, TPV, and ECO TPV

In comparison to conventional EPDM and thermoplastic vulcanizates (TPV and ECO TPV) evaluated in prior studies [18,19], both CCB and RCB EPDM formulations demonstrated enhanced chemical stability, particularly in terms of surface preservation and thermal response. Earlier investigations revealed that TPV-based materials, especially ECO TPV, were more prone to swelling, crack formation, and ionic release, with conductivity values in hot water extraction tests (HWETs) exceeding 8.0 µS/cm for ECO TPV [18].

In contrast, the present results indicate lower ionic leaching from the ECO EPDM systems. After 168 h of immersion in deionized water at 80 °C, RCB EPDM exhibited a conductivity of 7.1 µS/cm, while CCB EPDM remained below 5.5 µS/cm. Importantly, the 5 µS/cm conductivity threshold—commonly referenced in PEMFC component evaluation as an upper limit for ionic contamination [21]—was surpassed by RCB EPDM after 48 h, but only after 96 h for CCB EPDM. This behavior suggests a higher chemical resistance and slower ion release rate for the CCB formulation.

The significance of this threshold lies in its association with electrolyte stability and contamination risks within PEM fuel cells. Excessive ionic leaching from sealing materials can compromise membrane performance and accelerate degradation. As such, conductivity measurements serve as a useful indicator of material compatibility under long-term operating conditions.

Both EPDM variants remained under the 5 µS/cm threshold for at least the first 48 h, which is considered acceptable in PEMFC applications. However, the faster conductivity increase observed for RCB EPDM reflects a greater tendency toward ion release, potentially due to residual impurities or incomplete processing of the recycled filler. Despite this, both materials exhibited acceptable leaching performance over the test duration, supporting their relevance for environmentally friendly sealing applications, with CCB EPDM offering a more robust chemical profile under aqueous conditions.

### 4.2. CCB vs. RCB EPDM Formulations

Direct comparison between the two ECO EPDM formulations under identical acidic aging conditions revealed clear differences in chemical degradation trends. The RCB formulation exhibited higher ionic leaching, pH buffering effects (positive ΔpH values across all acid concentrations), and more pronounced surface degradation as evidenced by SEM. These outcomes are consistent with earlier observations on the mechanical deterioration of RCB EPDM, which included reduced tensile retention and increased swelling [20].

Interestingly, the inversion in leaching trends observed via HPLC-DAD—with RCB leaching more organic compounds at low acid concentrations (0.001–0.1 M), while CCB showed greater leaching at 1 M H_2_SO_4_—suggests filler-dependent thresholds in chemical resistance. This may be attributed to the higher degree of oxidative surface treatment and uniformity in CCB particles, which improves acid resistance under moderate conditions but could lead to the breakdown of secondary additives or interface degradation under severe acid exposure.

### 4.3. HPLC Peak Behavior and Wavelength Selection

The HPLC-DAD analysis was primarily performed at 230 nm, a wavelength chosen based on prior studies for its ability to capture unsaturated organics, including degradation byproducts such as alkylated aromatic compounds, diene oxidation fragments, or plasticizer residues [12,16]. Additional scans were conducted at other wavelengths (220–270 nm) but were omitted from the final discussion due to lower peak intensity or overlapping baseline interference.

Although compound identification was not possible in the absence of analytical standards or mass spectrometric data, the peak retention times (4.6–5.3 min) and UV absorbance profiles are tentatively consistent with leachates from hydrocarbon-based additives and residual polyaromatics, as reported in RCB-containing rubber systems [11,13]. This limitation is acknowledged, and future studies will focus on more advanced compound identification using HPLC-MS and NMR techniques.

### 4.4. Surface Morphology and Stability Implications

SEM analysis reaffirmed the chemical resilience of CCB EPDM, which maintained a smoother surface morphology even after 1000 h in 1 M H_2_SO_4_. In contrast, RCB EPDM developed surface cracks and increased porosity, aligning with earlier EDS observations of oxygen and sulfur enrichment [20]. These degradation markers support the hypothesis that the presence of residual oils and impurities in RCB facilitates local acid attack, reducing long-term durability.

### 4.5. Methodological Constraints and Future Directions

One key limitation of the present work is the lack of standardized calibration for HPLC-DAD analysis, which restricts interpretation to comparative peak area analysis without absolute quantification of leached compounds. Additionally, the current protocol did not include replicates across multiple batches, which would strengthen statistical confidence in the observed trends.

Future directions include the following:

- Preliminary validation of ECO EPDM materials under real or simulated PEMFC operating conditions—including mechanical cycling, humidity variations, and electrochemical stability—would strengthen the case for their implementation. Small-scale fuel cell testing or sealing simulation experiments should be considered.
- Identification and quantification of leached organics using HPLC-MS and GC-MS.
- Broader analysis of ion-specific leaching (e.g., Zn^2+^, Mg^2+^, and Si^4+^) using ion chromatography or ICP-OES.
- Long-term durability testing under dynamic PEMFC-like environments, including thermal–mechanical cycling and humidity variation.
- Exploration of additional sustainable fillers and compatibilizers to enhance RCB performance.
- Inclusion of thermogravimetric analysis (TGA) would allow a more comprehensive assessment of thermal stability and decomposition behavior.

## 5. Conclusions

1. CCB and RCB EPDM materials exhibit acceptable chemical stability under acidic conditions, with both maintaining functional integrity after prolonged exposure (1000 h) to sulfuric acid solutions at concentrations relevant to PEMFC environments.
2. Hot water extraction tests (HWETs) demonstrated reduced ionic leaching for both materials, with conductivity values remaining below the critical 5 µS/cm threshold for up to 48 h (RCB) and 96 h (CCB), supporting their suitability for fuel cell sealing applications.
3. RCB EPDM showed higher ionic release and greater pH buffering, indicating more active leaching of basic or contaminant species. In contrast, CCB EPDM presented a more stable pH profile and slower ion mobility, reflecting improved filler purity and chemical compatibility.
4. DSC measurements confirmed thermal stability in both systems, with minimal shifts in glass transition temperatures after acid aging. The slightly greater Tg depression observed in RCB EPDM suggests minor degradation of polymer chains or additive loss.
5. HPLC-DAD analysis revealed filler-dependent differences in organic leachate behavior, with RCB EPDM showing higher leaching at low acid concentrations and CCB EPDM exceeding RCB at high acidity (1 M H_2_SO_4_), indicating a concentration-dependent inversion in leaching mechanisms.
6. SEM analysis correlated well with chemical data, showing more severe surface degradation, porosity, and cracking in RCB EPDM after acid aging. CCB EPDM retained a smoother surface, highlighting improved structural resilience.
7. Overall, both ECO EPDM formulations are promising candidates for sustainable PEMFC sealing applications, but CCB EPDM consistently demonstrated superior performance in terms of chemical resistance, ionic stability, and surface preservation.

Future work should include compound identification of organic leachates via mass spectrometry, ion-specific leaching analysis (e.g., ICP-OES), and long-term dynamic testing under real PEMFC operating conditions to further validate the materials’ lifetime performance.

## Figures and Tables

**Table 1:** Initial mechanical and structural properties of the two EPDM compounds evaluated in this study, reproduced from [20].

Property	CCB EPDM	RCB EPDM
Tensile strength (MPa)	15	12
Elongation at break (%)	271	262
Shore A hardness	77	75
Microhardness (IRHD)	47	45
Crosslinking system	Peroxide	Peroxide
Filler type	Circular carbon black	Recycled carbon black

**Table 2:** Initial * and final pH values of sulfuric acid solutions (1 M, 0.1 M, and 0.001 M) after 1000 h of immersion at 90 °C for CCB and RCB EPDM materials, along with the corresponding pH change (ΔpH).

Material Type	Acid Concentration	Initial pH	Final pH	ΔpH (Change%)
CCB EPDM	1 M	0.10	0.00	−0.10 (−100%)
CCB EPDM	0.1 M	0.84	0.47	−0.37 (−44.0%)
CCB EPDM	0.001 M	2.24	1.41	−0.83 (−37.1%)
RCB EPDM	1 M	0.10	0.32	+0.22 (+220.0%)
RCB EPDM	0.1 M	0.84	1.00	+0.16 (+19.0%)
RCB EPDM	0.001 M	2.24	2.51	+0.27 (+12.1%)

* Initial pH values are based on reported measurements from prior work [19].

**Table 3:** HPLC-DAD results (230 nm) for samples extracted in 0.001 M H_2_SO_4_, 0.1 M H_2_SO_4_, and 1 M H_2_SO_4_ for 1000 h at 90 °C.

Material	Acid Concentration	Total Area (mAU * min)	% Change vs. CCB	Number of Peaks > 100 mAU * min
CCB EPDM	0.001 M	1872.06	−	5
RCB EPDM	0.001 M	2295.08	+22.6%	5
CCB EPDM	0.1 M	2271.69	−	6
RCB EPDM	0.1 M	2529.74	+11.4%	3
CCB EPDM	1 M	2980.14	−	4
RCB EPDM	1 M	1910.97	−35.9%	3

## Data Availability

The original contributions presented in this study are included in the article. Further inquiries can be directed to the corresponding author.
